# Emerging role of metagenomic next‐generation sequencing in infectious disease diagnostics: Clinical integration and future directions

**DOI:** 10.1002/mlf2.70078

**Published:** 2026-04-30

**Authors:** Tingting Fang, Feifei Yuan, Yu Chen, Na Li, Yao Zhang, Haixia Liu, Xingchen Liu, Qing Miao, Bijie Hu

**Affiliations:** ^1^ Department of Infectious Diseases, Zhongshan Hospital Fudan University Shanghai China

**Keywords:** bioinformatics pipelines, infectious disease diagnostics, metagenomic next‐generation sequencing (mNGS), public health surveillance, standardization of mNGS results

## Abstract

Infectious disease diagnostics has been transformed by metagenomic next‐generation sequencing (mNGS), an unbiased approach that detects bacteria, viruses, fungi, and parasites in a single assay. By sequencing all nucleic acids in a sample, mNGS overcomes the narrow detection scope and slow turnaround of conventional tests, substantially improving pathogen detection. In conditions such as meningitis/encephalitis, sepsis, and pneumonia, mNGS frequently identifies etiologies missed by routine diagnostic tests, thereby facilitating earlier pathogen‐directed therapy and, in selected settings, improving clinical management and outcomes. This approach is particularly valuable for immunocompromised, pediatric, and intensive care unit (ICU) patients with atypical infections. Currently, clinical mNGS workflows primarily rely on short‐read sequencing platforms (e.g., Illumina), whereas long‐read platforms (e.g., Nanopore, PacBio) offer advantages for rapid or high‐resolution applications. Optimized bioinformatics and stringent quality control are essential for reliable results. Beyond clinical diagnostics, mNGS provides valuable genetic data on antimicrobial resistance (AMR) and pathogen phylogeny, supporting public health and outbreak surveillance (e.g., wastewater monitoring and variant tracking). Current challenges include distinguishing colonization from infection, interpreting sequencing data quantitatively, and reducing cost and turnaround time. Looking ahead, emerging strategies such as targeted panels, rapid automated workflows, and host‑response integration are expected to further shorten time‑to‑result and improve diagnostic specificity. Parallel progress in ethical and regulatory frameworks remains essential to ensure responsible implementation. To support clinical adoption, a standardized framework for clinical interpretation of mNGS results, together with associated training, has been developed and implemented. Overall, mNGS is likely to become an increasingly important component of infectious disease diagnostics, with ongoing innovations expected to broaden its clinical and epidemiological impact.

## 1 INTRODUCTION

Infectious diseases remain a leading cause of global morbidity and mortality, and timely, accurate pathogen identification is imperative for effective treatment^1^. Traditional diagnostics, such as cultures, serology, and targeted PCR, are limited by their narrow detection scope and long turnaround time^2^. This diagnostic gap frequently leads to a prolonged and costly “diagnostic odyssey” for critically ill patients, where extensive, iterative testing fails to yield a definitive cause in up to 50% of meningitis/encephalitis or pneumonia cases^1^, ^3^, ^4^. Metagenomic next‐generation sequencing (mNGS) has emerged as a powerful approach to help overcome these limitations. By performing unbiased sequencing of all nucleic acids in a clinical sample, mNGS can detect bacteria, viruses, fungi, and parasites in a single assay without the need for any a priori assumptions^3^, ^5^, ^6^.

Early proof‐of‐concept cases (e.g., an unexplained neuroleptospirosis diagnosed by mNGS and reported in NEJM in 2014) highlighted the potential clinical value of mNGS^7^. Since then, mNGS has been applied across a wide spectrum of infections, yielding diagnoses that would have been missed by conventional methods^1^, ^8^, ^9^, ^10^, ^11^. Meta‑analytic and real‑world data consistently show superior etiologic yield versus conventional testing, with pooled sensitivity estimates around 70%–80% across syndromes^12^, ^13^, ^14^. In fever of unknown origin (FUO) cohorts, mNGS has approximately doubled diagnostic yield compared with culture‑based strategies. mNGS has also proven valuable for detecting rare pathogens (e.g., novel Yezo virus^15^ and Langya henipavirus^16^) and co‐infections (e.g., respiratory viral dual infections^17^ and multi‐pathogen HIV‐associated pneumonia^18^), thus enabling earlier targeted therapy and improved patient outcomes^17^, ^18^. A multicenter pneumonia trial suggested clinically meaningful benefit: incorporating mNGS reduced the time to clinical improvement (median 10 vs. 13 days)^19^.

mNGS also provides genomic data on antimicrobial resistance (AMR) and pathogen lineage, thereby supporting personalized therapy and outbreak tracking. However, challenges such as high cost, data complexity, and the lack of standardization currently hinder its adoption in routine clinical practice^20^, ^21^, ^22^. Beyond clinical diagnostics, mNGS holds substantial promise in public health surveillance. Environmental sequencing approaches (e.g., wastewater surveillance) have been shown to effectively detect emerging pathogens and monitor AMR, providing critical early‐warning systems^1^, ^23^. In the context of recent global health emergencies, rapid genomic surveillance via mNGS has enabled the identification of emerging viral variants, informing public health interventions and vaccine strategies^24^, ^25^. This review summarizes enabling technologies, clinical indications, decision impact, quality frameworks, and near‑term innovations that will shape clinical integration.

## 2 TECHNOLOGY ADVANCES OF MNGS

### 2.1 Evolution of sequencing platforms

High‐throughput short‐read sequencers like Illumina have been the workhorse of clinical mNGS, offering high accuracy and deep sequencing coverage. Illumina platforms enable parallel sequencing of hundreds of libraries, generating gigabases of data per run, which is essential for detecting low‐abundance pathogens^26^. This high‐throughput capacity also facilitates batch processing of multiple samples in a single run, a critical advantage for routine diagnostics. With optimized benchtop workflows, Illumina's sequencing can yield results in as fast as 14–24 h^26^. Ongoing improvements reinforce Illumina's position as the primary clinical mNGS platform, given its high accuracy, robustness, and scalability.

Another major player in second‐generation sequencing is BGI (MGI), which has introduced platforms like the BGISEQ and DNBSEQ series as alternatives to Illumina^3^, ^27^. These instruments utilize DNA nanoball (DNB) arrays and combinatorial probe‐anchor synthesis (cPAS) chemistry to achieve massively parallel short‐read sequencing^27^. For example, the MGISEQ‐2000 can generate ~1.4 terabases of data per run at approximately $10 per gigabyte^28^.

This cost‐effective performance, combined with data quality comparable to Illumina's, establishes BGI/MGI as a viable platform for clinical mNGS^27^. Indeed, a recent clinical evaluation found no significant differences in pathogen detection sensitivity or specificity between Illumina and BGI sequencers^26^.

Third‐generation sequencing platforms (such as Oxford Nanopore and PacBio) hold promise for expanding mNGS capabilities. Oxford Nanopore Technology (ONT) devices provide real‐time long‐read sequencing^29^, enabling ultra‐rapid diagnostics; for example, in bloodstream infections, a nanopore‐based targeted sequencing (NTS) assay delivered results in ~7 h from blood draw^30^. Nanopore's portability also permits bedside or field sequencing (e.g., in outbreaks)^31^. New high‐accuracy chemistries (e.g., Q20+ reads) and improved basecalling algorithms are narrowing this gap^32^, though further refinements and validation are needed for reliable clinical use. Consequently, current nanopore sequencing is predominantly applied in research or specialized situations (such as on‐site outbreak investigations or rapid sequencing for rare cases) rather than in routine hospital diagnostics.

PacBio HiFi sequencing, another long‐read technology, excels at generating high‐accuracy genomes useful for complete pathogen characterization (e.g., assembling plasmids or resistance elements) and discovering novel or unculturable organisms^33^. While PacBio's slower throughput and cost currently limit its routine clinical use, it has niche applications such as deciphering mixed infections or resolving repetitive genomic regions^34^, ^35^. Similar to ONT, the PacBio platform is presently utilized mostly in specialized research contexts rather than in standard diagnostics, as further improvements in throughput and cost‐efficiency are needed for broad clinical adoption.

Collectively, these sequencing platform advances have increased mNGS diagnostic sensitivity and reduced turnaround times^36^. Nonetheless, challenges persist in differentiating infectious agents from background nucleic acids and contaminants^37^. Approaches such as host DNA depletion and rigorous bioinformatic filtering are employed to improve specificity.

**Figure 1:**
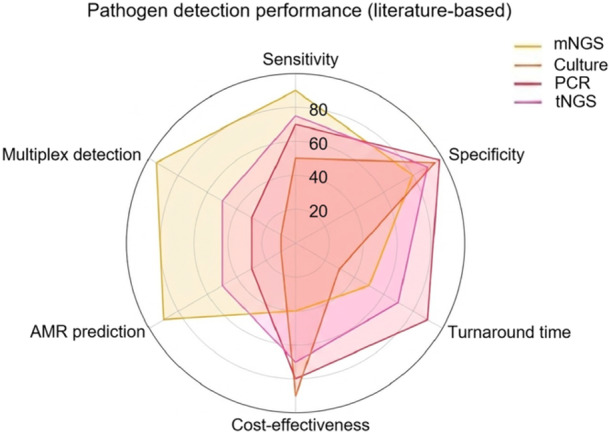
Comparative radar chart illustrating the performance characteristics of metagenomic next‐generation sequencing (mNGS) and conventional diagnostic methods. The chart is based on published data and compares four diagnostic modalities, that is, mNGS, culture, targeted PCR, and targeted next‐generation sequencing (tNGS), across six critical parameters: diagnostic sensitivity, specificity, turnaround time, cost‐effectiveness, antimicrobial resistance (AMR) prediction, and multiplex pathogen detection capability^1^, ^8^, ^36^, ^37^, ^38^, ^39^. It provides a schematic comparison of diagnostic characteristics across different modalities based on published data, highlighting the relative strengths and limitations of mNGS compared with culture, targeted PCR, and tNGS.

Figure 1 presents a comparative overview of mNGS performance metrics based on literature data. Literature‐derived estimates show that mNGS offers significantly higher sensitivity and multiplex detection capability than culture and PCR, particularly in syndromes like sepsis and central nervous system (CNS) infections^8^, ^38^. It also enables AMR gene profiling, which is largely absent from traditional tests^39^. However, mNGS still has longer turnaround times (24–48 h) and higher per‐sample costs, making it less cost‐effective than culture or PCR^1^, ^40^. Specificity remains high when appropriate contamination controls and interpretive thresholds are applied^30^, ^41^.

Thus, while third‐generation platforms offer exciting prospects for specific applications, such as point‐of‐care (POC) testing and complex genome assembly, the current bedrock of routine clinical mNGS diagnostics remains the high‐throughput, high‐accuracy short‐read sequencing provided by Illumina and BGI/MGI platforms.

### 2.2 Sample pre‑processing and host nucleic acid depletion

Clinical metagenomic specimens typically contain an overwhelming proportion of human nucleic acids (>95%–99%), necessitating preanalytical strategies to enhance microbial signal before sequencing. Optimized wet‑lab methods, including differential centrifugation, selective human DNA/RNA depletion (e.g., rRNA depletion and DNase treatment), and chemistry‑based host lysis, are routinely employed to reduce host background while preserving microbial nucleic acids^26^, ^42^, ^43^, ^44^. In a systematic evaluation of respiratory samples, a simple 15‑min centrifugation step achieved the highest viral‑read recovery among tested protocols^26^, ^45^, ^46^. Consistent with these findings, human rRNA depletion and DNase digestion have been applied in some laboratories for low‐titer or paucibacillary specimens to improve analytic sensitivity. These wet‑lab measures are complementary to downstream *in silico* host‑read subtraction and, when combined, materially increase the probability of detecting low‑abundance pathogens.

### 2.3 Bioinformatics pipeline optimization

Advances in bioinformatics are as crucial as sequencing hardware for maximizing mNGS utility. A key early step is host‑read subtraction, as clinical specimens (e.g., blood, cerebrospinal fluid [CSF], and bronchoalveolar lavage [BAL]) frequently contain >99% human sequences. *In silico* approaches enrich the microbial signal by aligning reads to a human reference genome (e.g., Genome Reference Consortium Human Build 38 [GRCh38]) and discarding matched fragments. They also utilize *k*‑mer‐based screening and complexity masking to remove residual human and artifactual sequences. Additionally, steps such as deduplication, quality trimming, and low‑complexity filtering further suppress background noise^26^, ^42^, ^43^, ^44^. The resulting non‑human read set provides a cleaner substrate for downstream taxonomic classification and assembly, improving sensitivity for low‑abundance pathogens and stabilizing quantitative estimates across samples.

Once sequences are generated, computational analysis must rapidly identify pathogens from millions of reads. Pipelines such as SURPI^+^ of the University of California, San Francisco (UCSF) and other in‐house workflows use curated reference databases (e.g., FDA‐ARGOS for high‐quality genomes) and tiered classification algorithms^26^. Reads are aligned to databases of viruses, bacteria, fungi, and parasites. Importantly, any unclassified reads undergo *de novo* assembly and translated alignment, enabling detection of novel or highly divergent organisms^26^. This approach was crucial for discovering new viruses—one assay detected 100% of tested viral pathogens by finding homology to related animal viruses, even after all human‐virus sequences were removed from the reference^26^. In addition, international organizations have begun releasing guidelines and standards. For instance, ISO 24420:2023 provides a standard for shotgun metagenomic data processing and quality evaluation to promote consistency across laboratories^43^. Adherence to these standards and robust validation (both “wet lab” and “dry lab”) is improving the reliability and reproducibility of mNGS results^43^, ^47^.

AMR gene prediction is now routinely integrated. Sequence data are cross‐referenced against resistance gene databases (CARD, ResFinder, and NCBI)^48^, ensuring that mNGS reports include not only the pathogen identified but also potential drug resistance. In one Chinese study of sepsis, nanopore sequencing correctly identified *mecA* and *vanA* resistance genes in methicillin‐resistant *Staphylococcus aureus* (MRSA) and vancomycin‐resistant *Enterococcus* (VRE) isolates, which showed an 80.6% correlation with phenotypic susceptibility test results^30^. However, genotypic resistance prediction should not be regarded as a direct substitute for phenotypic susceptibility testing, particularly for complex or incompletely understood resistance mechanisms.

Pipeline performance can vary, and benchmarking studies are guiding best practices. In one European Society for Clinical Virology (ESCV)‐led evaluation of 13 metagenomic virus pipelines, all pipelines detected high‐load viruses, but only 3/13 correctly identified low‐abundance pathogens in mixed infections^49^. This highlights the need for ongoing optimization, including improving detection algorithms for low‐level reads and enhancing the filtering of contaminants. Nonetheless, the gap between different analysis pipelines is closing as tools like Kraken2, MetaSPADES, and Kaiju are refined and combined, and as community proficiency testing (e.g., proficiency panels and external quality assessments) becomes available.

Beyond rule‐based algorithms, artificial intelligence (AI) and machine learning (ML) are being explored to improve mNGS analysis. AI can assist in differentiating true infection from contaminants by learning from large datasets of mNGS results and clinical correlates. For example, one group developed a “background knowledge base” of common commensals and environmental microbes and applied multidimensional thresholds (read count, abundance, and pathogenicity scoring), resulting in a 10% reduction in false positives^38^, ^50^. In the future, ML models could integrate host‐response patterns (e.g., transcriptional signatures of infection) with pathogen readouts to sequencing data to gauge the host immune response, potentially distinguishing between infectious and non‐infectious inflammation. Clinical uptake of mNGS hinges on workflow speed, reproducibility, and hands‑on simplicity. Three complementary strategies appear particularly important: (1) pre‑analytical automation to reduce hands‑on time and batch effects^26^, ^47^, ^51^; (2) compute‑side acceleration to compress analysis time without sacrificing accuracy^26^, ^32^, ^46^, ^48^, ^52^; and (3) integrated run governance to stabilize inter‑run performance^22^, ^26^, ^53^, ^54^, ^55^. In urgent indications (e.g., fulminant sepsis or encephalitis), a staged two‑tier approach is practical—deploying a rapid targeted panel or nanopore‑based read‑until protocol for an interim rule‑in within 6–12 h, while a parallel short‑read mNGS run completes within ~24 h to capture unexpected pathogens and AMR determinants^26^, ^30^, ^47^, ^51^, ^56^, ^57^. Across routine indications, these optimizations reduce end‑to‑end turnaround and improve lot‑to‑lot reproducibility, bringing mNGS closer to a standardized, clinically actionable diagnostic modality^47^, ^51^, ^53^.

### 2.4 Quality management and control

As mNGS moves from research into clinical laboratories, robust quality management systems (QMS) are paramount. Regulatory agencies have begun issuing relevant guidelines. For example, the US FDA's Breakthrough Device designation granted in 2023 to an mNGS‐based viral test underscores the need for rigorous validation^26^. That assay underwent extensive analytical validation, including limit of detection (LOD, ~543 copies/ml for viruses), precision, specificity, and stability testing. It achieved 93.7% overall accuracy versus gold‐standard PCR in a controlled study^26^. Laboratories developing mNGS as a clinical test must similarly establish performance characteristics in accordance with regulatory QMS guidelines (e.g., FDA's 21 CFR Part 820 or Clinical Laboratory Improvement Amendments [CLIA] of 1988 requirements). This includes running internal controls in every run. For example, an exogenous phage spike‐in and an External RNA Controls Consortium (ERCC) RNA mix are used as internal controls to monitor sensitivity and background^26^.

Standardization of protocols is being aided by international guidelines. The ISO 17822:2020 standard provides a “laboratory quality practice guide” for nucleic acid amplification‐based pathogen detection, outlining requirements for everything from specimen processing to result reporting. Alignment with ISO 17822 and CLSI MM guidelines may help ensure consistency in metagenomic test performance across clinical mNGS laboratories. Participation in external quality assessment (EQA) or proficiency testing programs is also on the rise. Although no fully established EQA for metagenomic diagnostics existed until recently, pilot inter‐laboratory comparisons have been conducted. For example, multiple labs sequenced the same mock infection samples to measure variability^53^, ^54^, ^55^. Such efforts will drive convergence toward “reference” pipelines and databases^58^, ^59^, ^60^. To address the need for standardized reporting, an expert‐consensus framework was developed to guide the clinical interpretation of respiratory mNGS results^61^, ^62^, ^63^, ^64^. The integration of host‐response information represents a cutting‐edge advance complementing direct pathogen detection. Because mNGS can capture host mRNA alongside microbial sequences (when RNA sequencing is performed), it offers a window into the host's immune response. Combining host transcriptomic analysis with mNGS has improved diagnostic accuracy in proof‐of‐concept studies, including tuberculosis (TB) meningitis and sepsis diagnostics, where high bacterial and viral detection accuracy has been reported^65^, ^66^. This integrated approach enhances differentiation between true infections and colonization, supporting clinical decision‐making even when pathogen counts are low^66^. Nevertheless, challenges remain: data interpretation complexity, contamination risks requiring stringent controls^11^, prolonged turnaround times, and higher costs compared to single‐pathogen tests. Ongoing multicenter trials and standardized guidelines aim to address these issues and establish best practices^19^, ^67^, further supporting the integration of mNGS into clinical diagnostic pathways^22^, ^68^.

To promote inter‐laboratory consistency for clinical metagenomics, the recently established harmonized standard for shotgun metagenomics (ISO 24420:2023) specifies rigorous requirements for data processing and quality evaluation (e.g., reference curation, contamination control, and reporting elements). Integrating ISO 24420 alongside ISO 17822 (nucleic‑acid amplification practice) provides a coherent quality framework spanning pre‑analytical handling, sequencing, and bioinformatic interpretation^43^. Adoption of these standards, together with CLIA/FDA QMS elements (method validation, version control, and internal/external controls), strengthens reproducibility and auditability across laboratories.

## 3 CLINICAL APPLICATIONS AND DECISION IMPACT

### 3.1 Broad clinical indications and evidence matrix

mNGS has been applied across a broad range of infectious disease settings. Here, we summarize key indications, highlighting evidence from multicenter studies and clinical trials, including several important studies led by Chinese investigators, that support the diagnostic value of mNGS.

#### 3.1.1 CNS infections

mNGS is particularly valuable in CNS infections (meningitis and encephalitis), where conventional diagnostics often fail. In a seven‐year UCSF study (>4800 CSF samples), mNGS identified a pathogen in 14.4% of cases and was the sole diagnostic method in 21.8% of confirmed CNS infections^1^. Notably, mNGS had much higher sensitivity (63.1%) than standard serologic tests (28.8%) for CNS pathogens^1^. Similarly, a multicenter cohort of 111 suspected CNS infection cases found that mNGS identified pathogens in 68.7% of patients, versus only 26.5% by culture (*p* < 0.0001)^69^, demonstrating a substantially higher detection rate. Chinese investigators have also made important contributions to the applications of mNGS in CNS infections. In 2024, a multicenter cohort study of adult meningitis/encephalitis across four Chinese hospitals compared unbiased mNGS with a targeted amplicon‐based NGS panel^56^. While broad mNGS detected 39 of 89 definite infections, the targeted panel identified 57 of 89, indicating a higher yield for known pathogens^56^. This suggests a complementary approach: using broad mNGS to catch unexpected or novel agents, and employ high‐sensitivity targeted sequencing for common pathogens. Importantly, in CNS infections of unknown etiology, mNGS has diagnosed treatable conditions such as herpes simplex virus (HSV) encephalitis, TB meningitis, and fungal meningitis that were missed by routine tests^9^, ^10^, ^11^. Furthermore, mNGS‐based diagnoses have shown important clinical utility. One study of 369 patients with CNS infections demonstrated that those tested with mNGS had more optimized antimicrobial regimens. Nearly half (49%) of these patients required a change in therapy based on the mNGS results, and they experienced reduced overall drug usage and slightly shorter hospital stays^70^. Clinical experience in both China and the United States supports the use of mNGS as a second‐tier test for CNS infections: if initial tests are unrevealing and the patient is deteriorating or has unusual features, CSF mNGS can be clinically decisive in selected cases. These findings underscore that, in CNS infections, mNGS not only increases diagnostic sensitivity but can also directly inform treatment decisions and potentially improve outcomes.

#### 3.1.2 Bloodstream infections and sepsis

Rapid pathogen identification in sepsis is critical, as each hour of delayed appropriate antibiotics raises mortality. Blood culture, the conventional standard, is slow and often negative if prior antibiotics were given. mNGS can detect bloodstream pathogens even when cultures are sterile. Han et al. conducted a prospective study of 387 sepsis patients in China using NTS and compared it against both mNGS and standard culture^30^. mNGS had a positivity rate of ~74.7%, more than double blood culture's 33.9%^30^. NTS yielded a slightly lower positivity (69.5%) that was not significantly different from mNGS, but it delivered results faster (often within 7 h)^30^. The range of pathogens detected by mNGS/NTS spanned typical bacteria (e.g., *Klebsiella pneumoniae* and *Staphylococcus aureus*), viral co‐infections (*Cytomegalovirus* [CMV] and Epstein‐Barr virus [EBV]), and fungi, many of which were missed by culture^30^. The study also noted that mNGS could simultaneously identify multiple pathogens in polymicrobial sepsis while also detecting genotypic resistance markers, enabling earlier optimization of therapy. Single‐center ICU experience has similarly suggested that mNGS may identify pathogens earlier than culture in selected cases, thereby allowing earlier de‐escalation of broad empiric antibiotics^63^. Han et al. further reported that the odds of pathogen detection were ~5‐fold higher with mNGS than with standard culture^30^. Real‐world emergency department data showed that adding mNGS increased diagnostic positivity from 19.5% to 50.5% in acute infections^71^. Plasma cell‐free DNA mNGS may further enhance sensitivity; in one selected cohort, it achieved 100% detection of bacteria/fungi in sepsis, outperforming the 88% sensitivity of cellular blood mNGS^72^. Combining plasma and cellular samples for mNGS maximizes detection while preserving specificity, facilitating timely identification of atypical or polymicrobial sepsis. However, careful interpretation remains crucial to differentiate true pathogens from contaminants^72^.

#### 3.1.3 Respiratory tract infections (pneumonia, TB and nontuberculous mycobacteria [NTM])

The respiratory system poses a challenge for mNGS due to the abundance of commensal flora and frequent colonization. Despite this, mNGS has proven valuable in diagnosing atypical pneumonias and pulmonary TB. By 2019, Chinese hospitals were routinely using mNGS on BAL fluid for pneumonia of unknown etiology. Recent clinical cohort studies have demonstrated that mNGS increased TB detection rates from ~35% with conventional tests to ~50% when applied to clinical samples^64^, ^73^, ^74^, especially in disseminated or extrapulmonary TB, where culture sensitivity is low. However, about half of the mNGS‐positive TB cases in our study had only 1–5 sequencing reads of *Mycobacterium tuberculosis*. This low abundance (likely reflecting the paucibacillary nature of TB and the organism's thick cell wall hindering DNA extraction) means that standard reporting cutoffs could miss true TB infections^64^, ^73^, ^74^. To address this, an interpretation framework that lowered the reporting threshold for TB/NTM and incorporated clinical context was developed, helping to ensure that these low‐abundance but potentially important reads are not dismissed^63^, ^64^, ^73^, ^74^. In common community‐acquired pneumonia (CAP), mNGS provides incremental diagnostic benefits, particularly by identifying viral or fungal pathogens not detected by routine tests. Notably, a multicenter randomized controlled trial (RCT) in China in severe ICU CAP provided level A evidence that mNGS can improve outcomes. Patients receiving mNGS (plus standard tests) had a higher 14‐day clinical improvement rate (62.0% vs. 46.5%) and achieved clinical stability ~3 days faster than those with standard tests alone^19^. In our experience, mNGS is especially useful for pneumonia in immunocompromised patients, often unveiling unexpected pathogens like *Pneumocystis jirovecii*, *Nocardia*, or viral co‐infections that alter management^63^. We are also investigating mNGS for NTM lung disease. Our ongoing study aims to determine if mNGS can detect NTM faster than culture and distinguish colonization from infection using read metrics. Early data suggest that although mNGS is less sensitive than concentrated sputum culture for NTM, it can identify disseminated or mixed NTM infections that would otherwise be missed^74^.

#### 3.1.4 Other emerging applications

mNGS is proving its versatility in many other infection syndromes. In endocarditis and prosthetic joint infections (PJIs), where pathogen yield from culture is low (especially after antibiotics or in biofilm‐associated infections), mNGS has detected pathogens in culture‐negative cases and uncovered polymicrobial infections, guiding surgical and antibiotic decisions^75^, ^76^. In ophthalmic infections, intraocular fluid mNGS has achieved high sensitivities (~92% in vitreous fluid and 85% in aqueous humor), vastly outperforming traditional diagnostics and improving outcomes in culture‐negative endophthalmitis^77^. For skin and soft tissue infections (SSTIs), mNGS identified pathogens in 67.7% of cases versus 35.4% by culture, detecting viruses and rare bacteria (e.g., *Vibrio vulnificus* and *Bartonella henselae*) that were missed by conventional tests^78^. Importantly, 41.7% of mNGS‐positive SSTIs had targeted antibiotic adjustments (vs. 3.8% for culture‐guided cases), underscoring the clinical utility^78^. Similarly, in bone and joint infections, adding mNGS significantly increased the detection rate of uncommon pathogens (21.6% vs. 10.2% with standard methods; *p* = 0.016)^76^, including atypical or fastidious organisms like *Tropheryma whipplei* and *Mycoplasma hominis*. The same study reported better outcomes in the mNGS‐guided group, such as shorter hospital stays, fewer antibiotic changes, and improved infection control rates^76^, highlighting that beyond diagnosis, mNGS can positively influence management and outcomes. Other reported uses of mNGS include fever of unknown origin (FUO), where it occasionally finds a treatable infection (e.g., *Bartonella* or zoonotic viruses) in otherwise cryptic cases^79^, and hospital outbreak investigations, where sequencing of environmental samples has been used to identify nosocomial infection sources.

**Figure 2:**
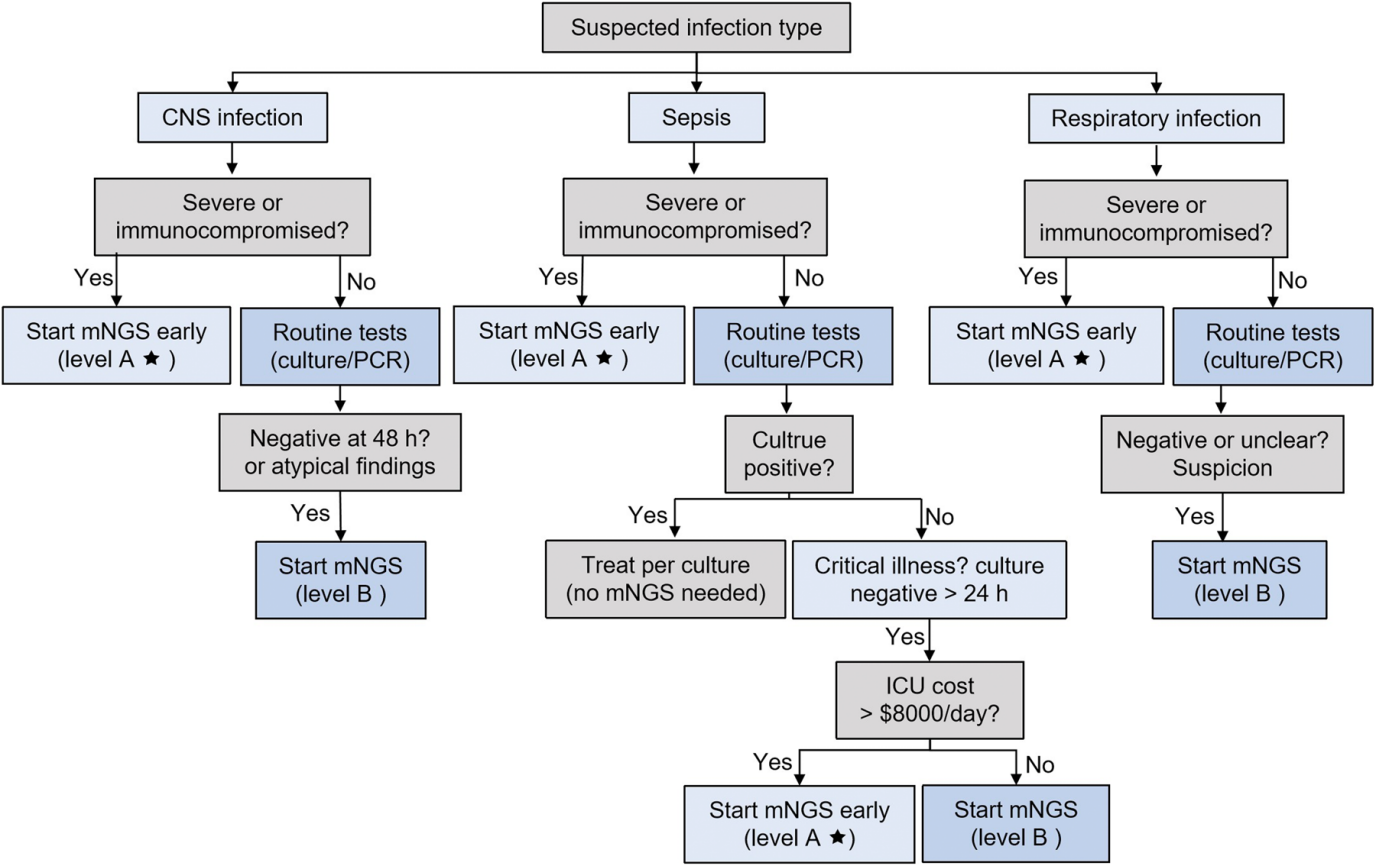
Clinical diagnostic algorithm incorporating mNGS. The decision tree outlines when to utilize mNGS based on infection syndrome (CNS infection, sepsis, respiratory infection), immune status, severity of illness, and outcomes of initial conventional tests. The algorithm emphasizes using mNGS selectively for cases where it adds most value, guided by evidence levels (A★, high‐quality evidence or strong consensus; B, moderate‐quality evidence). cCNS, entral nervous system.

A diagnostic pathway integrating mNGS into clinical practice is proposed (Figure 2). Typically, conventional diagnostics (cultures, antigen tests, and PCR panels) are performed first for rapid results. If these are negative or inconclusive and clinical suspicion for infection persists, especially in critical illness, mNGS is employed as a next‐line test^1^. In certain high‐risk scenarios (e.g., encephalitis or an immunocompromised host with sepsis), clinicians may initiate mNGS earlier in parallel with standard tests^1^, ^56^, ^67^. The decision algorithm emphasizes using mNGS when it is most likely to impact management, that is, when traditional methods fail to yield a diagnosis yet an infectious cause is strongly suspected^8^. Infectious disease specialist consultation is recommended to interpret mNGS results in context and to ensure appropriate follow‐up. By incorporating mNGS at strategic decision points, the overall diagnostic rate can be improved while minimizing unnecessary testing^1^.

Overall, these reports and others illustrate the broadening impact of mNGS across diverse infectious syndromes. High‐level reviews and consensus guidelines increasingly recognize mNGS as a powerful adjunct in pathogen identification and patient management, projecting an expanding role for clinical metagenomics in improving diagnosis and outcomes for complex infections. The clinical evidence supporting mNGS across major infection categories is summarized (Table 1). The applications of mNGS in CNS infections is supported by the highest level of evidence (level A), with pivotal studies demonstrating a substantial absolute increase in pathogen detection (~48%) and a high rate of treatment modification (~54%). Sepsis/BSI (level B) showed significant sensitivity improvement over cultures in a pivotal study by Kalantar et al.,^66^ while nontuberculous mycobacterial pulmonary disease (NTM‐PD) (level B) benefited from enhanced pathogen identification and targeted therapy^81^. Bone and joint infections currently have limited data (level C), with preliminary findings indicating high mNGS sensitivity in PJIs^82^. Pneumonia (level B) studies demonstrated moderate diagnostic and therapeutic benefits from BAL mNGS^83^. Table 1 highlights representative findings.

**Table 1:** Evidence matrix for clinical indications of metagenomic next‐generation sequencing (mNGS).

Infection type	Evidence level	Absolute increase (%, diagnostic yield vs. conventional methods)	Clinical applications	References
CNS infection	A	42.2	Gaining treatment modification rate of 49.%	[69, 70]
Sepsis/BSI	B	40.8	mNGS‐guided therapy halved the 7‐day mortality (from 8.6% to 5.2%)	[30, 67]
NTM‐PD	B	15	mNGS facilitated rapid differentiation of mixed infections to guide targeted therapy	[64, 73, 74]
Bone/joint infections	C	11.4	mNGS led to significantly “fewer antibiotic changes” and shorter hospital stays rather	[76]
Severe pneumonia/LRTI	A	A multicenter RCT demonstrated a reduction in the median time to clinical improvement from 13 to 10 days.	Gaining treatment modification rate of 77.4%	[80]

BSI, bloodstream infection; CNS, central nervous system; LRTI, lower respiratory tract infection; NTM‐PD, nontuberculous mycobacterial pulmonary disease; RCT, randomized controlled trial. Evidence levels: A, high‐quality evidence from multicenter studies or strong consensus; B, moderate‐quality evidence from prospective cohorts; C, limited data or preliminary retrospective findings.

### 3.2 Impact on treatment decisions and cost‐effectiveness

A critical question is whether mNGS results actually change clinical management in a meaningful way. Multiple studies—and our own experience—indicate that they do. One early mNGS pilot in challenging infection cases reported an approximately 15% increase in pathogen detection compared with standard tests, and more than 50% of cases had a change in clinical decision‐making based on the mNGS results^1^, ^19^. These changes included de‐escalating unnecessary broad‐spectrum antibiotics, adding coverage for an unexpected organism, or pursuing invasive diagnostic procedures when mNGS pointed to a specific focal infection. For example, in one case, mNGS of CSF identified *Taenia solium* (neurocysticercosis) when all other tests were negative, leading to targeted antiparasitic therapy and avoiding unwarranted empiric anti‐TB treatment. Such changes in clinical management may justify the use of mNGS in selected cases^2^, ^84^.

Multiple real‐world studies also show that mNGS results frequently prompt changes in patient management, particularly antimicrobial therapy adjustments. For instance, in a pulmonary infection cohort, 77.4% of patients with positive mNGS findings had their antibiotic regimens modified versus 25.7% of those with negative mNGS^80^. An orthopedic infection study further reported that patients who underwent mNGS had shorter hospital stays and fewer antibiotic‐related complications^76^. Similarly, another study noted that over half (57.7%) of patients in an mNGS‐guided group received adjusted antibiotic treatment within 2 days of admission, a much higher proportion than in the standard diagnostic group^85^. These mNGS‐guided adjustments allow more pathogen‐directed therapy and prompt de‐escalation of unnecessary broad‐spectrum antibiotics. Such changes can translate into better clinical outcomes. For example, in deep neck space abscesses, mNGS‐guided therapy achieved a significantly higher treatment success rate (relative risk ~1.22)^85^, along with trends toward shorter hospital and ICU stays (though these reductions were not always statistically significant)^85^, ^86^. Similarly, analyses in severe pneumonia showed that mNGS‐based treatment adjustments shortened average hospital stay by ~2.8 days and ICU stay by ~4.1 days^87^.

Importantly, early etiological diagnoses via mNGS may improve outcomes, including survival, in selected critical infections. A systematic review of severe pneumonia found that mNGS‐guided management was associated with significantly lower 28‐day mortality (~15% vs. 33% with conventional methods) and lower 90‐day mortality (~22% vs. 43%)^87^. Likewise, a large multicenter sepsis study reported that adding mNGS halved the 7‐day mortality (5.2% vs. 8.6%), although 28‐day mortality was similar between groups (possibly due to delayed interventions equalizing outcomes)^67^. These findings illustrate that mNGS can facilitate timely pathogen‐directed therapy in life‐threatening infections, thereby improving early survival and clinical cure rates. Conversely, by identifying noninfectious causes (or viral infections that do not need antibacterial treatment), mNGS can prevent unnecessary antibiotic exposure, indirectly improving patient safety and supporting antimicrobial stewardship.

Despite the higher upfront cost of mNGS, emerging evidence suggests it can be cost‐effective by reducing downstream healthcare expenditures and improving outcomes. A health economic analysis in China modeled mNGS use for suspected respiratory infections, estimating an average gain of 0.18 life‐years per patient and a net cost saving of ¥3069 ($450) per case when mNGS was added to traditional methods^28^. These savings were driven by optimized therapy (e.g., earlier appropriate treatment and avoidance of prolonged empiric broad‐spectrum antibiotics), resulting in shorter hospital stays and lower drug costs^28^. Real‐world data support this trend. For example, one comparative study found that total medical costs for patients managed with mNGS were significantly lower than for those managed with standard diagnostics, even including the sequencing test cost (median $3544 vs. $4823)^85^. This reduction was driven by shorter durations of ineffective therapy and faster clinical improvement in the mNGS‐guided group. Moreover, as the technology matures and sequencing becomes faster, the cost per test is gradually decreasing. Thus, although mNGS currently incurs a substantial upfront expense, its ability to streamline care by securing the correct diagnosis sooner, guiding targeted therapy, and averting unnecessary interventions may offset the initial cost and, in selected high‐impact scenarios, prove economically favorable^28^, ^29^.

Downstream cost savings should also be considered: a timely mNGS diagnosis can preclude many unnecessary tests or even surgeries. For instance, identifying a fungal pneumonia via mNGS might spare the patient an invasive lung biopsy. A US health‐economic analysis reported that while mNGS for FUO carries a high upfront cost, it is cost‐effective in cases where it finds a treatable infection because it averts a prolonged diagnostic odyssey^79^. Sequencing costs continue to decline, and when weighed against expenses like ICU care (~$3000/day) or ineffective treatments, a ~$500–$1000 mNGS test can be justified in the right clinical scenario^27^. In summary, mNGS has demonstrated a meaningful impact on treatment decisions by enabling earlier optimization of therapy, which has been linked to shorter hospitalizations, improved patient outcomes, and potential cost savings in appropriate contexts. However, broad use of mNGS in low‐acuity or likely self‐limited infections is not cost‐effective; current consensus is to deploy mNGS selectively for high‐impact cases, such as critically ill patients, elusive diagnoses where conventional tests failed, or potential outbreaks where rapid identification is urgent.

## 4 CHALLENGES OF MNGS

Despite its promise, mNGS introduces new challenges that clinicians and microbiologists must navigate.

### 4.1 Distinguishing infection from colonization

Accurate interpretation of metagenomic findings requires separating true infection from colonization or contamination, particularly for specimens from nonsterile sites (e.g., lower respiratory tract and gastrointestinal tract) where commensal taxa are expected^37^, ^88^, ^89^. A structured, semi‐quantitative approach integrating the following elements may be helpful: (i) read‑level metrics (unique reads, genome breadth/coverage, and microbe‑to‑human RPM ratios); (ii) organism‑specific prior probabilities and pathogenicity in the sampled compartment; (iii) internal/negative controls and laboratory “background” databases to down‑weight recurrent contaminants^1^, ^37^, ^88^, ^89^; and (iv) clinical correlates (host immune status, imaging, inflammatory markers, and response to therapy)^61^. For nonsterile specimens, higher reporting thresholds and dominance criteria (e.g., rank abundance and fold‑over‑background) reduce overcalling colonizers; for sterile compartments (e.g., CSF and blood), even low‑level signals of a plausible pathogen may be actionable after orthogonal confirmation (targeted PCR/culture or repeat sampling)^61^. In respiratory mycobacterial disease and invasive fungal disease, tailored thresholds and compartment‑specific rules (“Zhongshan standard”) improve sensitivity without materially inflating false positives by coupling minimal read counts with strong clinical–radiologic concordance^23^, ^61^. Finally, when mNGS detects multiple taxa, we advocate a tiered report (dominant/minor/trace) accompanied by interpretive comments that explicitly state the likelihood of causality and suggest confirmatory tests, thereby aligning laboratory output with bedside decision‑making^61^.

### 4.2 Handling unexpected or novel pathogens

mNGS's ability to detect rare and previously unknown organisms means clinicians may be confronted with unfamiliar names. In recent years, cases of obscure infections have surged: *Chrysosporium* lung infection in an immunocompromised host, *Bartonella vinsonii* endocarditis in a patient with culture‐negative fever, and a new circovirus in a transplant patient—all found by mNGS^90^, ^91^, ^92^. Similarly, a other study highlights mNGS identification of previously obscure viruses such as the Potosi virus implicated in human encephalitis^1^. These findings often require expert consultation and careful literature review to determine their clinical relevance and therapeutic implications. Furthermore, truly novel pathogens (e.g., a novel virus or bacterium) present a reporting dilemma. The pipeline might assemble a sequence that clearly indicates a new species; reporting “unknown organism” has limited value to clinicians. One strategy is to report the closest known relative (e.g., novel parvovirus related to B19 detected) to give a hint of the pathogen class. Ongoing curation of databases and inclusion of newly discovered respiratory viral genomes are needed so that novel agents can be recognized faster^52^. Continuous curation and expansion of comprehensive reference databases are critical for rapid recognition of emerging pathogens; major commercial mNGS services currently utilize databases referencing over 21,000 microbial genomes^93^. International collaboration in sharing novel sequences via public genomic databases is also part of the solution.

### 4.3 Interpreting clinical significance and integrating results

Unlike a simple positive/negative test, mNGS generates an abundance of data—often requiring expert interpretation. Clinicians must consider questions like: Is the identified organism plausible as the cause of the patient's disease? Does it fit the clinical picture (e.g., *E. coli* in CSF mNGS could be a contaminant if the patient lacks signs of Gram‐negative meningitis)? Are multiple organisms suggestive of a polymicrobial process (e.g., abscess), or could they be artifactual? To aid in this, mNGS reports now often include additional information such as the reads mapped, genome coverage, and even charts of microbial abundance. For instance, one structured reporting approach classifies organisms with >30% of microbial reads as dominant, those with 5%–30% as minor, and those with <5% as trace, together with comments on whether each organism is known to cause the infection type. This structured reporting framework has implemented in clinical practice and shared across multiple hospitals as a practical interpretive template^64^. It mirrors efforts elsewhere (the UCSF group published a similar proposed reporting format in 2022^65^). Additionally, international guidelines such as ISO standards (ISO 20397 and ISO 23420 series) and expert consensus publications are emerging to standardize mNGS result interpretation^22^. Another inherent challenge lies in conveying diagnostic uncertainty, particularly because DNA‐based mNGS cannot differentiate between viable organisms and residual nucleic acids. To address this, advanced laboratory reports increasingly incorporate standardized interpretive caveats. For instance, a report might state: “*Streptococcus pneumoniae* DNA was detected; immediate clinical correlation is strongly advised, as this organism frequently represents transient nasopharyngeal carriage rather than invasive infection in non‐sterile respiratory specimens.” This type of comment helps clinicians gauge whether to act on a finding.

AI tools and ML algorithms are increasingly explored to facilitate interpretation by integrating host‐response profiles and microbial data. Recent studies using AI‐driven classification have reported >95% accuracy in distinguishing infection from non‐infectious inflammation, suggesting potential value in streamlining clinical decision‐making in complex cases^93^, ^94^. In summary, interpretation is both an art and a science, requiring standardized frameworks, case‐by‐case judgment, and sometimes further testing (such as targeted PCR to confirm a low‐level finding). Building multidisciplinary teams (infectious disease physicians, microbiologists, and bioinformaticians) for mNGS result review is one practical strategy to help ensure that reports are accurate and clinically meaningful.

Despite these challenges, they are surmountable with experience and guidelines. Each difficult case is an opportunity to refine interpretation criteria. As usage grows, collective knowledge (through published case series and consensus guidelines) will continue to improve clinicians' ability to discriminate true infection from background noise in mNGS results. Our vision is that, in the near future, interpreting an mNGS report will become as routine as interpreting a culture result, with the help of robust standards and perhaps AI‐driven decision support, highlighting likely pathogens versus bystanders.

### 4.4 The clinical significance of a negative mNGS report

A negative mNGS report should be interpreted cautiously and cannot be regarded as definitively exclusionary, particularly because its predictive value varies substantially by sample type. Its predictive value varies dramatically by sample type. For instance, recent studies have highlighted surprisingly high false‐negative rates in CSF (59.6%) and other sterile body fluids (69.8%), whereas negative results from plasma demonstrate higher accuracy in ruling out infection (72.0%)^95^. Such discrepancies may arise from several factors, including pathogen loads below the assay's limit of detection, the presence of difficult‐to‐lyse organisms (e.g., fungi with thick cell walls), or pathogens whose genomes are absent from reference databases. Therefore, the clinical weight of a negative mNGS result must be carefully calibrated against the pre‐test probability of infection, the specific sample source, and the overall clinical context.

## 5 DEVELOPMENT ROADMAP OF MNGS

### 5.1 Emerging technologies and enhanced workflows

The next horizon for infectious disease diagnostics involves faster, more targeted sequencing workflows and integration of host data. One important trend is the rise of targeted NGS (tNGS) panels as a complement to unbiased mNGS. By capturing or amplifying dozens of specific pathogens of interest, tNGS achieves higher depth on those targets, improving sensitivity and turnaround. We saw in the Chinese encephalitis study that a panel of 50 common CNS pathogens outperformed broad mNGS in picking up known causes^56^. Similarly, for sepsis, targeted nanopore sequencing matched mNGS's breadth but delivered results in hours^30^. In the future, hybrid approaches will likely dominate: for example, do a quick tNGS for a defined pathogen set (results in <6–12 h) in parallel with an mNGS run. If tNGS yields an answer, one can act immediately; if not, the mNGS data (ready in ~24 h) might reveal an unexpected pathogen. This two‐tier model balances speed and open‐ended detection.

Another area of development is ultra‐rapid library preparation and sequencing. Efforts are underway to compress the sequencing timeline to just a few hours. For example, researchers are exploring direct RNA sequencing for RNA viruses, enzymatic library prep kits that work in 15 min, and nanopore adaptive sampling (which can enrich reads of interest in real‐time). A scenario in which samples obtained in the emergency department are sequenced on admission and yield actionable results within hours is becoming increasingly plausible. Recent efforts have demonstrated preliminary pathogen identification within 30 min of sequencing initiation, completing total workflows within approximately 6–8 h, significantly accelerating diagnosis compared to traditional workflows^57^.

The concept of a “<6 h mNGS” has been demonstrated in research settings for certain infections (e.g., sequencing CSF in meningitis within 4–6 h using nanopore). To achieve this clinically, every link of the chain (extraction, sequencing, and analysis) is being optimized. Integrated, automated platforms combining extraction, library preparation, sequencing, and bioinformatics analysis have emerged, reducing turnaround time and manual error; such systems have shown robust clinical performance, achieving sensitivities approaching 87% and specificities near 100% for viral pathogens^26^, ^51^. Automation and POC devices will play a role: modular instruments that perform sample‐to‐sequence on a benchtop (e.g., cartridge‐based mNGS systems in development^51^) could simplify workflows and reduce the need for specialized technical skills. POC mNGS platforms are advancing rapidly, notably Oxford Nanopore's MinION, which provides portable, real‐time sequencing. MinION has successfully been used for pathogen identification during major viral outbreaks in resource‐limited settings^96^, and demonstrated high diagnostic performance even in culture‐negative meningitis cases^29^. These applications highlight MinION's potential in urgent and resource‐limited scenarios; however, its routine use in hospital diagnostics remains limited by modest throughput and the need for standardized workflows to ensure consistent results. Meanwhile, PacBio's platform, though less portable, offers high‐fidelity sequencing (>99% accuracy), effectively resolving complex genomic structures, AMR genes, and virulence factors, enhancing comprehensive pathogen characterization^97^. However, the platform's longer run time and high cost currently limit its suitability for rapid POC testing; accordingly, PacBio is predominantly utilized for comprehensive genomic analyses in specialized laboratories rather than as a front‐line clinical diagnostic tool.

### 5.2 Clinical integration strategies (from lab to bedside)

For mNGS to realize its potential, it must be effectively integrated into clinical practice on a broad scale. One strategy is to develop regional mNGS reference labs or networks. Not every hospital can maintain an in‐house metagenomics lab (given costs and required expertise), but a hub‐and‐spoke model can allow hospitals to send samples to specialized centers with results returned swiftly. In China, several regional centers (e.g., BGI in Shenzhen, and Shanghai center at Zhongshan Hospital) now receive samples from secondary hospitals, run mNGS, and report back within 24–48 h. To facilitate this, standardization of sample handling and transportation is needed (stabilization tubes, cold chain, etc., to preserve nucleic acids). Telemedicine platforms can integrate results such that clinicians in outlying hospitals can consult with experts on interpretation.

Another integration aspect is developing hospital guidelines and clinical pathways for when and how to use mNGS. Early hospital‐level protocol for infectious disease mNGS testing have established clear diagnostic criteria. One representative recommendation states that mNGS should be considered after three days of negative routine tests in life‐threatening infections or performed earlier when an atypical pathogen is suspected (e.g., culture‐negative encephalitis)^62^, ^63^. Such institutional pathways may also facilitate reimbursement decisions and set expectations regarding turnaround time and downstream clinical action. Many hospitals are now forming mNGS review committees (similar to antibiotic stewardship committees) that review requests to ensure appropriate use and then help interpret results. This not only optimizes patient care but also collects valuable data on mNGS performance across different departments, feeding back into institutional knowledge.

Reporting standards represent another integration focus. We have touched on the importance of how results are presented. Ideally, reports from different labs should have a common format such that a physician reading an mNGS report from lab A versus lab B can easily find the key information. International efforts (like the CDC's Sequencing Quality Workgroup) are aiming to publish recommended reporting elements: for example, listing organisms in order of descending abundance, providing a measure of confidence or statistical significance for each, and clearly flagging any known pathogens of critical concern (like *Neisseria meningitidis* or *Yersinia pestis*, which have public health implications). Our respiratory mNGS reporting framework represents one step toward this goal and has been shared as a practical template to promote more consistent reporting language across centers in China^63^, ^64^. Consistent reports also facilitate data pooling for research (so cases can be compared across studies more easily).

Finally, a critical piece of integration strategies is educating and training clinicians. Even a technically robust mNGS assay will have limited clinical impact if front‐line clinicians do not trust or understand how to use it. As part of an effective clinical integration strategy, regular hospital‐wide case conferences can be conducted to demonstrate how mNGS resolves diagnostic dilemmas. Furthermore, incorporating mNGS principles into infectious disease fellowship training programs is highly recommended. Such efforts demystify the technology and underscore that, while advanced, mNGS is just another diagnostic tool—one that clinicians can learn to use judiciously. This approach addresses a cultural hurdle: initial clinical skepticism regarding the utility of “genomic tests” for infections is gradually overcome as objective evidence and successful diagnostic cases accumulate.

### 5.3 Standardization and future directions

As the field matures, standardization will be the linchpin that ensures mNGS tests reliable and comparable across different settings. One priority is establishing reference standards and controls that all labs can use. For example, synthetic microbial communities with known compositions (e.g., DNA from five pathogens at set concentrations spiked into human plasma), can serve as proficiency test materials^55^. Initiatives are underway to create such reference materials–akin to how labs calibrate viral load assays with WHO standards. Regulators may eventually require mNGS labs to use these controls in validation and participate in EQA proficiency panels annually to maintain quality. Recent results from an EQA in Europe^49^ showed significant inter‐lab variability in detecting certain low‐level pathogens, highlighting the need for improvement. Through iterative EQAs, methods will likely converge (labs that underperform can learn from those with better results).

From a regulatory perspective, the debate between handling mNGS as a single laboratory‐developed test (LDT) versus multiple component tests is ongoing. In the United States, the FDA is moving toward overseeing high‐complexity LDTs like mNGS. This could mean that commercially developed mNGS kits or pipelines might require FDA clearance/approval, which in turn demands multi‐center trials and demonstration of clinical validity. We anticipate that in the next five years, the first FDA‐approved mNGS diagnostic kits will emerge—possibly one for CNS infections and one for respiratory infections. These would come with standardized reagents, software, and reporting templates, making it easier for hospital labs to adopt mNGS without building everything in‐house. Our lab is preparing for this by ensuring our pipeline is CLIA‐ready; we document all standard operating procedures (SOPs), perform CLIA validation (accuracy, precision, and analytical sensitivity/specificity) similar to any new clinical assay, and have an extensive quality management process (including routine run audits and periodic update assessments). We have also obtained PCR laboratory accreditation and ethical approvals to run mNGS clinically, paving the way to seek formal designation as a clinical test under national policy.

At an international level, collaboration will fuel standardization. Data‐sharing consortia being discussed internationally might allow pooling thousands of mNGS cases^22^. Automated interpretation systems could also be developed from such pooled data; for example, cloud‐based services where raw mNGS data from anywhere in the world are analyzed against a continuously learning database to provide a preliminary report. Of course, these raise data governance and ethical issues (who owns and can access sequencing data? how to protect patient privacy?), but technically they could dramatically accelerate standardization (everyone using the same analytic engine).

Lastly, the future direction of mNGS standardization will also involve clinical practice guidelines. We expect professional societies (e.g., Infectious Diseases Society, Chinese Medical Association Infectious Disease Branch) to issue guidelines or best practice statements on the use of metagenomic sequencing. These would cover indications, how to integrate results, and even suggested treatment algorithms following an mNGS diagnosis (for instance, an algorithm for CNS infections might say: if mNGS positive for pathogen X, treat accordingly; if negative, consider brain biopsy, etc.). Our team's contribution to publishing the first expert consensus on respiratory mNGS provides a template for such guidelines^64^. This expert consensus (the 'Zhongshan standard') has subsequently helped to standardize respiratory mNGS interpretation and reporting practices across numerous clinical centers in China^62^, ^63^, ^64^. We are now participating in drafting a broader guideline for mNGS in clinical infectious diseases in China. Internationally, as RCT evidence grows (like the pneumonia trial), guidelines will incorporate mNGS into care pathways (e.g., an upcoming Surviving Sepsis campaign update might include mNGS as an option when initial cultures are negative in septic shock)^19^, ^22^.

Beyond clinical settings, mNGS has substantial potential for public health surveillance, offering unprecedented capabilities for early detection and monitoring of emerging pathogens and AMR. Metagenomic sequencing of environmental samples—such as wastewater, air, and vector populations—can act as an early‐warning system, capturing genetic signals from pathogens before clinical outbreaks become evident^98^. Integrating a “One Health” approach, mNGS can bridge human, animal, and environmental data streams, rapidly detecting zoonotic diseases and emerging health threats^98^.

Moreover, mNGS significantly enhances the monitoring of AMR gene prevalence and dissemination. Metagenomic analysis of wastewater samples allows simultaneous surveillance of numerous resistance determinants and resistant bacteria, providing critical real‐time data for public health interventions and resistance trend tracking^99^. Such comprehensive methods complement traditional resistance tracking systems, helping mitigate AMR spread more effectively^99^.

**Figure 3:**
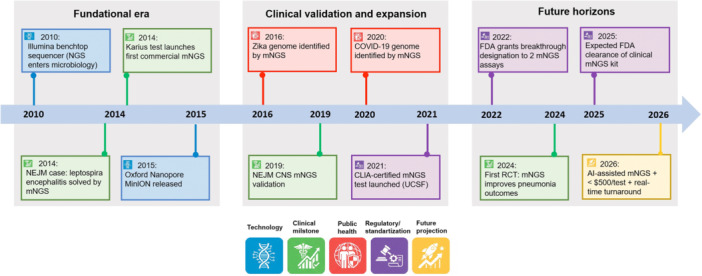
This timeline details pivotal technological breakthroughs, regulatory advancements, and clinical validation studies shaping mNGS from initial proof‐of‐concept to its anticipated widespread clinical adoption. It highlights key milestones such as the first successful mNGS diagnosis (2014), large‐scale clinical validations, and expected future advances in rapid testing and artificial intelligence (AI) integration. CLIA, Clinical Laboratory Improvement Amendments; FDA, Food and Drug Administration; NEJM, New England Journal of Medicine; RCT, randomized controlled trial.

The coming years are poised to refine and expand mNGS in clinical medicine. Figure 3 illustrates key milestones in mNGS development and adoption over the past two decades, anticipated near‐future advances, and timeline of metagenomic NGS in infectious disease diagnostics, highlighting notable milestones and future directions. Key developments include the advent of next‐generation sequencing in 2004–2005, which laid the groundwork for metagenomic approaches^84^; the first clinical mNGS success in 2014 (identification of an unsuspected pathogen by unbiased sequencing in a critically ill encephalitis patient)^1^; the implementation of routine diagnostic mNGS services by 2016 (e.g., at academic centers)^1^; and the accumulation of evidence by 2019 through multicenter studies and case series confirming mNGS utility across multiple infectious syndromes^1^, ^100^. The COVID‐19 pandemic in 2020 underscored the importance of rapid genomic surveillance and pathogen discovery, further accelerating interest in metagenomic methods. By 2024, demonstrations of clinical impact (such as the seven‐year UCSF study) have solidified mNGS as a credible diagnostic tool^1^. Looking ahead, innovations in the early 2020 s are focusing on reducing turnaround time (with reports of <24‐h sequencing workflows)^8^ and enhancing automation and interpretation. These advancements are expected to drive broader adoption in the coming years and to further integrate mNGS into routine infectious disease diagnostics in appropriate clinical settings. In summary, the developmental roadmap involves continued advances in both technology and clinical practice: new sequencing and AI tools will expand capabilities, while parallel efforts in standardization, validation, guideline development, and public health surveillance will help ensure that these capabilities translate into reproducible diagnostic performance and meaningful clinical benefit across diverse healthcare settings.

## 6 CONCLUDING REMARKS

In conclusion, mNGS has evolved from an emerging concept into a practical clinical tool, marking an important advance in infectious disease diagnostics. To further support its clinical integration, a useful framework may be summarized as the 3 Ts: technology, translation, and training.

In technology, continued innovation in sequencing platforms, bioinformatics pipelines, and automation is needed to make mNGS faster, more cost‐effective, and even more accurate. This includes embracing third‐generation sequencing for real‐time results and integrating host‐response analysis to augment pathogen detection. Significant progress in this field involves developing enhanced bioinformatic filters to reduce false positives and optimizing sampling methodologies to improve diagnostic yield. Continued refinement of these analytical tools, along with the broad sharing of multi‐center clinical data, will be crucial for establishing global standardization in mNGS diagnostics^63^, ^64^.

Broader clinical integration of mNGS will also depend on factors such as health insurance reimbursement, standardized protocols, and its integration with traditional diagnostics like culture and PCR. Recent health economic analyses suggest that incorporating mNGS into reimbursement frameworks may improve patient access and potentially reduce overall healthcare costs by enabling earlier targeted therapy and shorter hospital stays^78^, ^101^. Establishing standardized protocols and rigorous quality control criteria is critical to ensure reliability and reproducibility across diverse clinical laboratories^102^. Importantly, mNGS does not replace traditional methods but rather complements them. Combined diagnostic approaches have demonstrated superior performance, especially in complex clinical scenarios^22^. For example, randomized studies in ICU settings have shown that combining mNGS with routine diagnostics significantly shortens the time to clinical improvement compared to standard methods alone (median 10 vs. 13 days), underscoring its additive value^19^.

Certain patient populations, particularly immunocompromised individuals, children, and critically ill ICU patients, may derive substantial benefits from wider mNGS adoption. Immunocompromised patients often present with atypical infections that conventional diagnostics miss; in this population, mNGS markedly improves diagnostic accuracy, guiding targeted therapy and improving outcomes^101^. Similarly, pediatric studies demonstrate that mNGS offers superior sensitivity for detecting bloodstream infections and severe pneumonia in children, enabling timely therapeutic interventions^103^. Critically ill ICU patients also benefit from mNGS's rapid, comprehensive pathogen detection, which can reduce mortality by enabling faster identification and appropriate antimicrobial adjustments^19^.

In translation, bridging the gap between bench and bedside is paramount for the clinical adoption of mNGS. This requires establishing standardized protocols (like the “Zhongshan standard”)^63^, ^64^, conducting clinical trials (such as the severe community‐acquired pneumonia [SCAP] RCT in ICU patients^86^) to generate high‐level evidence, and securing regulatory approvals that validate mNGS as a clinical test. The recent publication of regional guidelines and consensus documents underscores the ongoing transition of mNGS research into standardized clinical practice. We envision a future where every major hospital has either in‐house or network access to mNGS and where it is incorporated into routine diagnostic algorithms for appropriate scenarios.

In training, the human factor, such as educating clinicians, microbiologists, and data scientists, is the glue that holds everything together. We must train a new generation of professionals (“microgenomic” specialists) fluent in both clinical infectious diseases and sequencing analysis. To achieve this, the establishment of dedicated training centers for high‐throughput sequencing in clinical settings is essential, with a strong emphasis placed on cross‐disciplinary learning among physicians and laboratory scientists.

On a global scale, mNGS holds substantial potential for global health, especially in resource‐limited settings. Despite infrastructure and cost barriers, initial studies in low‐ and middle‐income countries have demonstrated the feasibility of mNGS for pathogen surveillance and outbreak control^104^. With appropriate investment in portable technologies like nanopore sequencing and sustainable training programs, mNGS could significantly strengthen global infectious disease preparedness, surveillance, and health equity^96^, ^105^.

In essence, the path forward for metagenomic sequencing in medicine hinges on responsible innovation. By focusing on “3 T” while proactively addressing ethical considerations, we can firmly embed mNGS into routine clinical practice as a reliable, equitable, and invaluable tool. The work by our team and many others has laid a strong foundation, from constructing one of China's first clinical mNGS platforms to authoring consensus standards, and these contributions will continue to propel the field. As mNGS becomes more widely available, we anticipate a future where no patient remains undiagnosed due to technical limitations. Instead, mNGS will enable clinicians to identify pathogens swiftly and accurately, ushering in an era of precision infectious disease therapy and improved patient outcomes worldwide. The vision of turning the unknown into the known—transforming the “mystery” fever into a defined treatable infection—is rapidly becoming reality, fulfilling the promise of metagenomics as a cornerstone of 21st‐century infectious disease diagnostics.

## Supporting information

Supplementary Table 1: Performance Comparison of mNGS and Conventional Diagnostic Methods for Infectious Diseases.
